# Forecasting the Path of a Raccoon Rabies Epidemic

**DOI:** 10.1371/journal.pbio.0030115

**Published:** 2005-03-01

Rabies recently hit the national headlines when a Wisconsin teenager survived after showing full-blown symptoms. Even more remarkable, the girl—who was bitten by a bat—recovered after receiving a novel therapy, since doctors felt her case was too advanced for the standard rabies inoculations to work. Rabies is nearly always fatal if not treated immediately, and continues to pose a serious public health threat. Though most rabies fatalities in the United States stem from bat bites, far more people are treated for raccoon rabies.

A new strain of raccoon rabies started spreading throughout the eastern United States in the mid-1970s, after raccoons caught in Florida were released along the West Virginia–Virginia border to replenish hunting stocks. Some of the imports carried a rabies variant that caused an outbreak in local populations and has been steadily expanding ever since. In 1990, raccoons topped the list of most often reported rabid mammal.[Fig pbio-0030115-g001]

**Figure pbio-0030115-g001:**
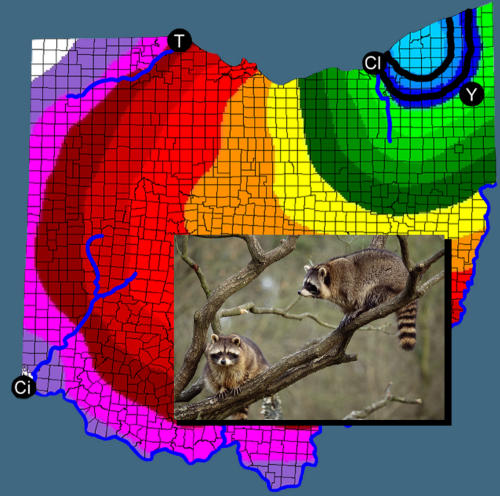
Predicting the spread of raccoon rabies across Ohio

Controlling this re-emerging public health threat depends on predicting the spatial dynamics of the disease—where new outbreaks might occur and how the virus might spread. Toward this end, Leslie Real and colleagues work on probabilistic simulation models that calculate the effects of various factors, such as local transmission rates between townships, ecological barriers to transmission, and long-distance “translocation” rates between townships. (The deliberately released Florida raccoons were one such translocation, but raccoons have also been known to hitch rides on garbage trucks.) As reported elsewhere, these models previously accurately reflected rabies spread in both Connecticut and New York. In a new study reported in *PLoS Biology*, Real and colleagues apply their model to the likely spread of rabies in Ohio—a potential gateway for spread throughout the Midwest—and find that raccoon rabies could spread throughout the state in just three years.

One strategy for limiting rabies spread is to establish vaccine corridors by distributing vaccine baits—vaccine doses hidden in fishmeal—to wild raccoons. This *cordon sanitaire* strategy limited rabies in Ohio to sporadic cases from 1997 until 2004, when a rabid animal was detected—11 kilometers beyond the buffer zone—in northeastern Ohio. The authors had previously shown that local transmission was significantly reduced when townships were separated by geographical barriers—the Connecticut River in Connecticut and the Adirondack Mountains in New York. In modeling the likely transmission path in Ohio, the authors incorporated the likely effect of Ohio's five major rivers on transmission from local points along the Pennsylvania or West Virginia border.

Given Ohio's topography (three of its rivers run along the southern and eastern border) and a single point of emergence in the northeast, the authors adjusted their simulations to estimate the potential impact of translocations. Even without the occasional garbage truck ride, because of the lack of ecological barriers in central Ohio, the simulations predict that rabies will spread far faster in Ohio than in New York and Connecticut.

Factoring in those garbage truck rides, the scenario is considerably bleaker: rabies would take just 33 months to spread across central Ohio—compared to 48 months to cross the much smaller state of Connecticut—and cover the state in 41 months. This transmission rate—100 kilometers/year—significantly surpasses previous estimates, which range from 30 to 60 kilometers/year. The potential for such rapid spread, if unchecked, “is quite alarming,” the authors warn. But they also point out that the path of a real epidemic would likely fall somewhere between these two scenarios, given the unpredictable nature of translocations. The authors also simulated potential breech points in the vaccine corridor and found that the Ohio and Muskingum rivers halted viral advance initially. But a raccoon can certainly cross a bridge when the opportunity arises, so any delays would likely be temporary.

Given the unpredictable nature of rabies transmission—challenging efforts to identify potential leaks in vaccine corridors and sites of dispersal—the authors' simulations provide a valuable resource for anticipating alternate outbreak scenarios and preparing multiple game plans to prevent or contain them. They also indicate the best sites for establishing a new vaccine barrier. And given how fast raccoon rabies could spread, Real and colleagues make a strong case that halting its western march depends on a strategy based on early detection and high-powered intervention programs—a sensible approach for any infectious disease.

